# Deformities of the Nose

**Published:** 1867-02

**Authors:** Chas. F. Taylor

**Affiliations:** New York


*Deformities of the Nose. Operation to Repair a Broken Down Septum Narium.* By CHAS. F. TAYLOR, M.D., of New York.

As a contribution to what is conveniently called "minor surgery," though often requiring more careful study and skill than more dangerous and imposing operations, the following case is given, in the hope that its suggestions may be of service:—

H., of Detroit, Michigan, a lad fourteen years old, was brought to me in November, 1865, for treatment for complete occlusion of the right nasal passage. I found the septum narium broken and doubled up, the fold of the cartilage projecting into and filling up the right nasal passage. It was supposed to have been done by a fall on the nose when about four years old. The cartilage was hypertrophied and unyielding, and did not seem susceptible of modification by any means short of an operation. There were so many objections to the ready operation of excising the extruded portion of the septum—the danger of troublesome hemorrhage from the pituitary membrane, the tardy healing of the exposed cartilage, the sensitiveness of the cicatrized surface, as well as the frequent failure of the operation itself to give the desired relief—that I was induced to devise an operation which seemed to promise important advantages.

Preparing myself with a very small knife, having a thick back, awl point and short cutting edge, I introduced it through the integument which covers the inferior border of the septum, and, carefully guiding it *between* the mucous membranes by one finger inserted in the nostril, the septum was cut longitudinally through and through in several places along the bent portion, till the tumor became soft and compressible. On withdrawing the knife, the wound, which was not more than a mere prick of an awl through the skin, immediately closed up, and not half a dozen drops of blood were lost. The mucous membranes were not pierced in any place, the knife having been kept carefully between them. A clamp of hard rubber had been previously prepared exactly fitting the nasal passages, one leg of which was then introduced into each nostril and screwed tightly together, compressing the tumor, straightening up the septum, and bringing the mucous membranes covering the cartilage near together. The hypothesis was that the operation and compression would excite a certain amount of inflammation in the parts, and that as in subcutaneous tenotomy, the wound being surrounded on all sides by healthy tissue, healing by first intention, or at least rapid reparation, might be expected. It was necessary, however, that the clamp should be kept on long enough to secure the desired result, and not retained so long as to cause sloughing and possible disaster. The clamp was removed in eighteen hours after its application and reapplied less tightly for twelve hours longer, when, on removing it, the septum remained in its natural upright position. Healing by first intention had taken place, and both nasal passages were entirely unobstructed. There was slight nasal discharge, which soon subsided, and the cure was complete. During the whole time, even when the clamps were on the patient suffered no pain or the least in convenience.

In this case, the septum being depressed and partially doubled on itself, there was little actual deformity, save a somewhat flattened appearance of the nose; but the voice was injured by the stopping up of one nostril, and it was for this that an operation was desired by the parents. But why might not the same or a similiar operation be just as available for the removal of the common deformity, when the septum and consequently the nose is twisted to one side? In such a case I would suggest cutting the septum cartilaginum *obliquely* through, antero-posteriorly, near its base—keeping, as in this case, the knife carefully between and not piercing the mucous membranes—and then adjusting a clamp, as in this instance.

